# FeSe_2_-BiSe_2_-CoSe_2_ Ternary Heterojunction for Efficient Hydrogen Evolution Reaction Under pH-Universal

**DOI:** 10.3390/ma19020430

**Published:** 2026-01-22

**Authors:** Lili Guo, Yang Cui, Qiusheng He, Kankan Liu

**Affiliations:** 1 School of Materials Science and Engineering, Taiyuan University of Science and Technology, Taiyuan 030024, China; guolili1@tyust.edu.cn; 2 Shanxi Key Laboratory of Coordinated Management and Control for Environmental Quality, School of Environment and Resources, Taiyuan University of Science and Technology, Taiyuan 030024, China; 2020052@tyust.edu.cn; 3 School of Environment and Safety Engineering, North University of China, Taiyuan 030051, China

**Keywords:** heterojunction, FeSe_2_-BiSe_2_-CoSe_2_, hydrogen evolution reaction

## Abstract

The construction of heterostructures has been recognized as an effective strategy for enhancing material activity and stability. Herein, a ternary heterojunction FeSe_2_-BiSe_2_-CoSe_2_ was synthesized via a hydrothermal selenidation reaction. The significant electronegativity difference between Bi and Fe/Co triggers charge transfer within the FeSe_2_-BiSe_2_-CoSe_2_ lattice. Furthermore, the abundant pore structure of FeSe_2_-BiSe_2_-CoSe_2_ provides efficient pathways for electron diffusion, significantly enhancing the HER catalytic kinetics. Results demonstrate that FeSe_2_-BiSe_2_-CoSe_2_ exhibits outstanding HER activity in both acidic and alkaline media. In 0.5 M H_2_SO_4_, it exhibits an overpotential of only 44 mV with a Tafel slope of 108 mV dec^−1^. In 1 M KOH, the corresponding overpotential is 188 mV, with a Tafel slope of 45 mV dec^−1^ at 10 mA cm^−2^. This study constructs electron-rich active sites through electronic structure regulation, providing valuable insights for designing low-cost, high-performance transition metal selenide HER catalysts.

## 1. Introduction

Humanity currently faces the dual crises of energy scarcity and environmental degradation. As non-renewable resources, the consumption of available fossil fuels far exceeds their production rates. Consequently, developing sustainable clean energy has become a critical research focus for resolving the energy crisis [1,2]. Hydrogen energy, recognized as a green and clean energy source due to its high energy density and low-carbon, pollution-free attributes, is extensively studied as a fossil fuel alternative [3]. Large-scale hydrogen production holds the potential to facilitate a low-carbon transition in energy structures. Electrolytic hydrogen production from water represents a highly promising sustainable hydrogen generation technology [4]. The development of low-cost, high-performance hydrogen evolution catalysts holds significant importance for enhancing the efficiency of water electrolysis [5].

Conducting the HER under pH-universal is crucial for evaluating a catalyst’s practical potential and advancing the real-world application of hydrogen energy technologies. The actual industrial scenarios for water electrolysis hydrogen production are not confined to ideal alkaline or acidic media but are diverse. Therefore, a catalyst that performs excellently only at a single pH value will have its application scope severely limited. Developing HER catalysts with full pH activity means that the same material can adapt to multiple technological pathways. From a scientific perspective, investigating a catalyst’s performance and mechanisms across the entire pH range provides deeper insights into the reaction kinetics and stability mechanisms of its active sites under different species supply environments. This approach offers a more comprehensive evaluation of the catalyst’s intrinsic properties and structural advantages compared to single-pH studies [6].

To date, the most effective HER electrocatalysts have been precious metal Pt-based catalysts. However, precious metals exhibit dissolution, agglomeration, and instability during water splitting, while their cost and scarcity severely constrain their development [7]. Transition metal-based electrocatalysts have garnered significant attention due to their abundant reserves and tunable electronic structures. Among these, binary alloy compounds, such as Fe-, Co-, and Bi-based catalysts, exhibit high intrinsic activity in both acidic and alkaline media [8]. However, issues such as low conductivity and limited active sites require further optimization. Atomic doping (with non-metallic atoms) can effectively modulate the material’s electronic structure, expose active sites, and enhance mass transfer processes, thereby improving catalytic performance [9]. We compared the sulfides [10], selenides [11], oxides [12], nitrides [13], and phosphides [14] of transition metals (Table 1).

Fe, as a multivalent transition metal, possesses flexible electronic configurations and efficient electron donor–acceptor sites, enhancing the semiconductor properties of single selenides and reducing the electron transport energy barrier. Fe-based transition metal diselenides (MSe_2_, M=Fe, and Co) exhibit diverse chemical properties as metallic three-dimensional electrons increase, with FeSe_2_ and CoSe_2_ demonstrating numerous chemical characteristics [15,16]. Hydrothermally synthesized FeSe_2_ reveals enhanced active sites for ion and electron transfer. Se-rich vacancies in FeSe_2_ can yield superior HER performance through optimized M-O and M-H bonding. The 2p orbital electron configuration of transition metal Co enables precise regulation of the d-band center position within the catalytic active site [17]. The introduction of Co modulates the d-band center towards the Fermi level through intra-lattice electron interactions, optimizing hydrogen adsorption energy (ΔG_*H_) and thereby enhancing the intrinsic catalytic activity of the active site. During the HER activity process in CoSe_2_, the Co cation acts as the hydrogen anion (H^−^) receptor center, while the anion serves as the proton (H^+^) receptor center. The H_2_O molecule binds to the locally positively charged Co center, facilitating greater electron localization on the Se atom. This promotes the adsorption of H_ads_ on the Se anion and the conversion of H_ads_ to H_2_ molecules, thereby enhancing the electron transport rate on the catalyst surface [18].

The electronegativity of Bi exhibits significant differences compared to Fe and Co. This disparity triggers directional charge transfer within the lattice, reducing the electron density around Bi atoms to form electron-deficient sites [19]. Conversely, Fe and Co become electron-rich active sites due to electron enrichment. This accelerates charge separation and transport during catalytic reactions, preventing reaction inhibition caused by electron accumulation at the active sites [20].

Based on the aforementioned theory, this study prepared a ternary heterojunction FeSe_2_-BiSe_2_-CoSe_2_ catalyst via a one-step hydrothermal synthesis method. Results demonstrate that FeSe_2_-BiSe_2_-CoSe_2_ exhibits outstanding HER performance in both acidic and alkaline solutions. In a 1 M KOH alkaline solution and 0.5 M H_2_SO_4_ acidic solution, the overpotential at a current density of 10 mA cm^−2^ is merely 188 and 44 mV, respectively, with corresponding Tafel slopes of 45 and 108 mV dec^−1^. Its outstanding performance is attributed to the incorporation of Bi, which alters the electronic structure surrounding Fe and Co, transforming it into an electron-rich configuration. This effectively accelerates electron transfer and enhances the material’s electrical conductivity. Furthermore, characterization techniques such as SEM have revealed a substantial porous structure within the ternary heterojunction FeSe_2_-BiSe_2_-CoSe_2_. These richly developed pore structures generate additional active sites for HER initiation and progression, accelerating the kinetics of the catalytic reaction. A method for preparing transition metal-based ternary heterojunction selenides has been proposed.

## 2. Materials and Methods

**Figure 1:**
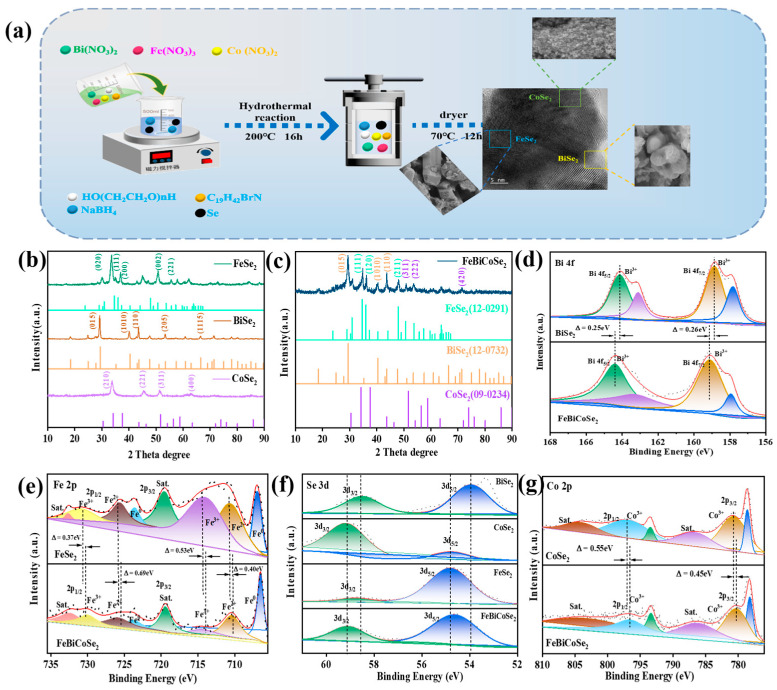
(**a**) Synthesis of FeSe_2_-BiSe_2_-CoSe_2_, (**b**) XRD patterns of FeSe_2_, BiSe_2_, and CoSe_2_, (**c**) XRD pattern of FeSe_2_-BiSe_2_-CoSe_2_. High-resolution XPS spectrum for (**d**) Bi 4f, (**e**) Fe 2p, (**f**) Se 3d, (**g**) Co 2p of FeSe_2_, BiSe_2_, CoSe_2_, and FeSe_2_-BiSe_2_-CoSe_2_.

We dissolved Co(NO_3_)_2_·6H_2_O, Bi(NO_3_)_2_·5H_2_O, Fe(NO_3_)_2_·9H_2_O (1 mmol each), and surfactants (HO(CH_2_CH_2_O)nH and C_19_H_42_BrN) in a water–ethanol mixed solvent to form Solution A. We dissolved Se and NaBH_4_ in the same solvent to form Solution B. After mixing, the reaction was conducted hydrothermally at 200 °C for 16 h. The product was obtained by centrifugation, washing, and drying at 60 °C. For comparison, BiSe_2_, CoSe_2_, and FeSe_2_ were synthesized under identical conditions using only the corresponding single metal salt precursors (Figure 1a).

Electrochemical measurements were performed in an electrochemical workstation (CHI 660E) using a standard three-electrode system with 1 M KOH as electrolytes, carbon rods, and Ag/AgCl and Hg/HgO electrodes as counter electrodes and reference electrodes, respectively.

## 3. Results

### 3.1. Characterization

X-ray diffraction (XRD) analysis was employed to investigate the chemical composition of BiSe_2_, FeSe_2_, CoSe_2_, and FeSe_2_-BiSe_2_-CoSe_2_, as shown in Figure 1b,c. The diffraction peaks at 30.21°, 35.01°, 37.03°, 50.83°, and 55.69° in FeSe_2_ correspond, respectively, to the (020), (111), (200), (002), and (221) crystal planes. Its lattice constants were calculated as a = 4.857 Å, b = 5.786 Å, and c = 3.587 Å (Table S1), which are in close agreement with JCPDS No. 12-0291 (a = 4.815 Å, b = 5.808 Å, and c = 3.599 Å) [21]. For BiSe_2_, the diffraction signals generated by its (015), (1010), (110), (205), and (1115) crystal planes appear at 29.23°, 40.15°, 43.56°, 53.43°, and 66.54°, respectively, in the XRD pattern. This is essentially consistent with the standard card (JCPDS No. 12-0732). Calculations yielded lattice constants of a = 4.151 Å, b = 4.151 Å, and c = 28.666 Å (Table S2), exhibiting slight deviations from the reference values (JCPDS No. 12-0732, a = b = 4.133 Å and c = 28.620 Å) [22]. In the XRD pattern of CoSe_2_, a series of characteristic diffraction peaks are observed. The peaks at 33.74°, 45.87°, 51.35°, and 62.91° correspond to the (210), (221), (311), and (400) crystal planes of CoSe_2_ (JCPDS No. 09-0234), respectively. Its lattice constants a = 5.874 Å, b = 5.874 Å, and c = 5.874 Å (Table S3) are essentially identical to the reference values (JCPDS No. 09-0234, a = 5.858 Å). This indicates the successful synthesis of FeSe_2_, BiSe_2_, and CoSe_2_ via a hydrothermal reaction [23].

With the incorporation of metal ions with differing atomic radii—Fe, Bi, and Co—into the same crystal lattice, atoms of varying sizes experience interdiffusion. Within the XRD pattern of FeSe_2_-BiSe_2_-CoSe_2_ (Figure 1c), diffraction peaks attributable to FeSe_2_, BiSe_2_, and CoSe_2_ can be distinctly observed. The diffraction peaks of FeSe_2_ at 34.89°, 36.22°, and 48.12° correspond to the (111), (120), and (211) crystal planes, respectively. For CoSe_2_, peaks at 51.01°, 53.78°, and 71.51° correspond to the (311), (222), and (420) crystal planes. The leftward shift in diffraction peaks corresponding to the FeSe_2_ (111) and CoSe_2_ (311) planes results from lattice stretching and broadening of diffraction peaks due to the incorporation of Bi, which possesses a larger atomic radius [24]. The diffraction peaks of BiSe_2_ at 29.35°, 40.44°, and 43.73° correspond, respectively, to the (015), (1010), and (110) crystal planes. The rightward shift in the diffraction peaks for the (015), (1010), and (110) planes in BiSe_2_ arises from the substitution of Bi atoms in the original lattice by Fe or Co atoms, which possess smaller atomic radii. Due to the significantly smaller atomic radii of Fe and Co compared to Bi, lattice contraction occurs. According to Bragg’s equation (nλ = 2dsinθ), this subsequently leads to a shift in the diffraction angle. FeSe_2_ is characterized by lattice parameters of a = 4.857 Å, b = 5.786 Å, and c = 3.587 Å; BiSe_2_ is characterized by parameters a = b = 4.151 Å and c = 28.666 Å; and CoSe_2_ is characterized by parameters a = b = c = 5.874 Å, which are essentially identical to their corresponding standard reference values; XRD analysis confirmed the successful formation of FeSe_2_-BiSe_2_-CoSe_2_ [25].

X-ray photoelectron spectroscopy (XPS) was employed to analyze the chemical composition and valence states of the samples via full-spectrum analysis. As depicted in Figures S1 and S2, the full spectra of BiSe_2_, FeSe_2_, CoSe_2_, and FeSe_2_-BiSe_2_-CoSe_2_ reveal the presence of four elements: Bi, Co, Fe, and Se. Figure 1d displays the high-resolution XPS spectra of the Bi 4f orbitals for BiSe_2_ and FeSe_2_-BiSe_2_-CoSe_2_. The peaks at binding energies of 164.13 and 158.86 eV correspond to the Bi 4f_5/2_ and Bi 4f_7/2_ orbitals in BiSe_2_, respectively, while the peaks at 164.38 and 159.12 eV can be attributed to the Bi 4f_5/2_ and Bi 4f_7/2_ orbitals in FeSe_2_-BiSe_2_-CoSe_2_, respectively. Both exhibit characteristics of Bi^3+^, with binding energies shifted to the left by 0.25 and 0.26 eV, respectively. This indicates a reduction in electron density around Bi, potentially arising from electron transfer due to differences in electronegativity between neighboring atoms within the alloy. This facilitates accelerated electron redistribution during electrocatalytic reactions, thereby enhancing electrocatalytic activity [26].

In Figure 1e, the high-resolution Fe 2p spectra of FeSe_2_ and FeSe_2_-BiSe_2_-CoSe_2_ reveal characteristic peaks at 731.04 and 714.31 eV in FeSe_2_, corresponding, respectively, to the 2p_1/2_ and 2p_3/2_ orbitals of Fe^3+^. Relative to FeSe_2_, the 2p_1/2_ (730.67 eV) and 2p_3/2_ (714.05 eV) orbitals of Fe^3+^ in FeSe_2_-BiSe_2_-CoSe_2_ exhibit rightward shifts of 0.37 and 0.26 eV, respectively. This results in increased electron density and enhanced binding capacity for surrounding electrons. The characteristic peaks of Fe^2+^ in FeSe_2_ on the 2p_1/2_ and 2p_3/2_ orbitals are located at 725.81 and 710.65 eV, respectively. Compared to FeSe_2_, the Fe 2p_1/2_ peak (725.48 eV) corresponding to Fe^2+^ in FeSe_2_-BiSe_2_-CoSe_2_ exhibits a rightward shift of 0.33 eV; the Fe 2p_3/2_ peak (710.25 eV) also shifts to the right by 0.40 eV [10]. The increased electron density around the Fe atom reduces its binding energy, confirming a change in the chemical environment surrounding the Fe atom, with a significant increase in its electron cloud density [8].

Figure 1f compares the Se 3d orbitals in different materials. The Se 3d_3/2_ binding energies in BiSe_2_, CoSe_2_, and FeSe_2_ are 57.79, 58.39, and 58.05 eV, respectively, with corresponding Se 3d_5/2_ binding energies of 53.73, 54.42, and 54.50 eV, all corresponding to the Se^2-^ state. In FeSe_2_-BiSe_2_-CoSe_2_, the binding energies of Se 3d_3/2_ and Se 3d_5/2_ further increase to 59.10 and 54.78 eV, respectively, with a reduction in electron density around Se. Electrons flow towards the transition metals Fe, Bi, and Co. Compared to FeSe_2_-BiSe_2_-CoSe_2_, the Se 3d_3/2_ and Se 3d_5/2_ peaks in BiSe_2_, CoSe_2_, and FeSe_2_ exhibit a shift towards lower binding energies, indicating that Se atoms also undergo significant electronic regulation within multivalent alloys. This demonstrates enhanced electron transfer between Fe, Bi, Co, and Se, which favorably contributes to elevated electrocatalytic activity [19].

Figure 1g displays the high-resolution XPS spectra of the Co 2p orbitals for CoSe_2_ and FeSe_2_-BiSe_2_-CoSe_2_. The characteristic peaks of Co^3+^ in FeSe_2_-BiSe_2_-CoSe_2_ exhibit shifts of 0.55 and 0.45 eV to the right, relative to those in CoSe_2_ (Co 2p_1/2_: 797.03 eV and Co 2p_3/2_: 780.75 eV). The corresponding binding energies are 796.58 and 780.30 eV. This phenomenon indicates that the electron density around Co increases, enhancing its metallic character and consequently improving the material’s electrical conductivity [27].

In FeSe_2_-BiSe_2_-CoSe_2_, it is not a simple unidirectional charge transfer that occurs, but rather a complex charge rearrangement. Crucially, as part of the matrix, the electron density of Bi and Se decreases, while that of the embedded transition metals Fe and Co increases. Alloying fundamentally alters the chemical environment of Se, whose electronegativity may differ from that in binary selenides, thereby influencing the charge distribution across the entire lattice. Acting as catalytic active centers, Fe and Co receive greater electron feedback from the alloy system, becoming electron-rich centers that facilitate electron redistribution. The electron-rich Fe and Co centers optimize their d-band electronic structure, aiding in the stabilization of certain low-valent intermediates during reactions or modulating redox potentials, thereby enhancing intrinsic activity. The Bi-Se matrix framework, with its tightened electronic structure, enhances the material’s metallic character and conductivity, ensuring rapid charge transport during the catalytic process [28].

**Figure 2:**
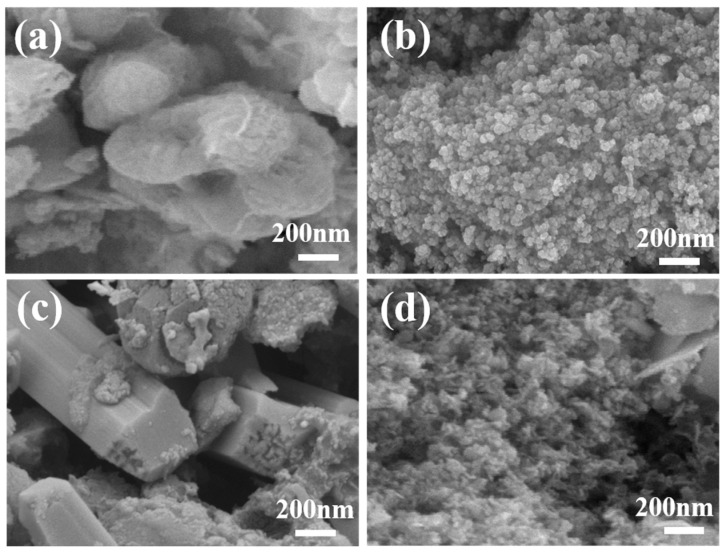
SEM images of (**a**) BiSe_2_, (**b**) CoSe_2_, (**c**) FeSe_2_, and (**d**) FeSe_2_-BiSe_2_-CoSe_2_.

Scanning electron microscopy (SEM) images of various materials reveal the morphology and microstructure of four catalysts: BiSe_2_, CoSe_2_, FeSe_2_, and FeSe_2_-BiSe_2_-CoSe_2_. As shown in Figure 2a and Figure S3a,b, the BiSe_2_ morphology comprises nanospheres with a relatively smooth surface, each nanosphere being relatively independent and measuring 1.5 μm in diameter. The prepared CoSe_2_ comprises irregularly shaped, uniformly sized particles with a relatively smooth surface, facilitating uniform electrolyte contact and ion diffusion (Figure 2b and Figure S3c,d). The FeSe_2_ nanorods synthesized in Figure 2c interconnect to form a relatively regular arrangement, uniformly dispersed across the substrate’s surface. This configuration yields a high specific surface area, providing numerous active sites conducive to the progression of electrocatalytic reactions (Figure 2c and Figure S3e,f). As shown in Figure 2d and Figure S3g,h, BiSe_2_ nanospheres are attached to FeSe_2_ nanorods, with CoSe_2_ nanoparticles present in the gaps between them, thereby forming a ternary FeSe_2_-BiSe_2_-CoSe_2_ heterojunction. The presence of CoSe_2_ prevents BiSe_2_ and FeSe_2_ from forming larger agglomerates, thereby increasing the surface area of the catalyst exposed to the electrolyte.

**Figure 3:**
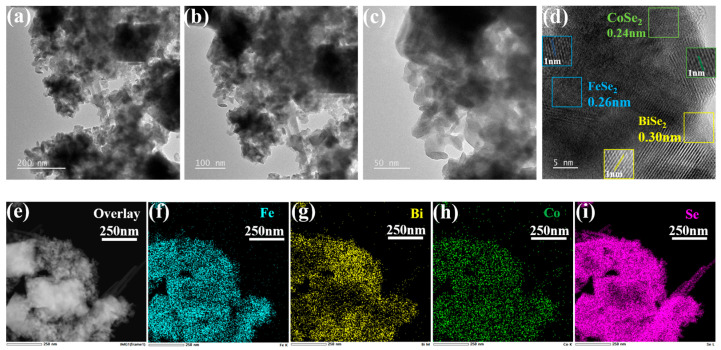
(**a**–**c**) TEM images, (**d**) HRTEM image, and (**e**–**i**) EDS element mapping images of FeSe_2_-BiSe_2_-CoSe_2_.

To investigate the polycrystalline properties of FeSe_2_-BiSe_2_-CoSe_2_, detailed analysis of its microstructure was conducted using TEM. Figure 3a–c display transmission electron microscopy images of the FeSe_2_-BiSe_2_-CoSe_2_ composite at different magnifications. Consistent with scanning electron microscopy characterization, this ternary heterojunction clearly shows BiSe_2_ nanospheres attached to FeSe_2_ nanorods, with CoSe_2_ particles visible in the gaps between BiSe_2_ and FeSe_2_. This arrangement provides additional active sites for chemical reactions, thereby enhancing the HER activity of FeSe_2_-BiSe_2_-CoSe_2_. The coexistence of multiple crystalline faces enables synergistic catalytic effects, optimizing reaction pathways. Further HRTEM observations (Figure 3d) reveal that the doped phases within the blue, green, and yellow boxes correspond to BiSe_2_, CoSe_2_, and FeSe_2_, respectively. Their lattice stripe interplanar spacings are 0.302 nm, 0.242 nm, and 0.259 nm, corresponding successively to the (110) plane of BiSe_2_, the (210) plane of CoSe_2_, and the (200) plane of FeSe_2_. This aligns with the corresponding crystal planes identified by XRD, confirming the coexistence of these three crystalline phases within the FeSe_2_-BiSe_2_-CoSe_2_ composite material and further corroborating its polycrystalline nature. Furthermore, energy-dispersive spectroscopy (EDS) analysis results, as shown in Figure 3e–i, reveal uniform distribution of Fe, Bi, Co, and Se elements throughout the composite material.

### 3.2. HER Performance in 0.5 M H_2_SO_4_

**Figure 4:**
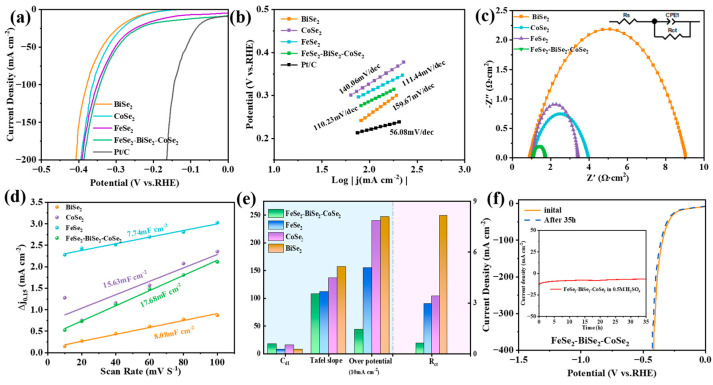
HER performance of BiSe_2_, CoSe_2_, FeSe_2_, and FeSe_2_-BiSe_2_-CoSe_2_ in 0.5 M H_2_SO_4_. (**a**) LSV curve, (**b**) Tafel slope, (**c**) Nyquist plot, (**d**) C_dl_ curve, (**e**) performance comparison bar chart of BiSe_2_, CoSe_2_, FeSe_2_, and FeSe_2_-BiSe_2_-CoSe_2_, and (**f**) LSV curve of FeSe_2_-BiSe_2_-CoSe_2_ after 35 h operation in 0.5 M H_2_SO_4_ and i-t curve during continuous 35 h electrolysis (inset).

With the structural, compositional, and basic physical profiles of the catalysts established, we then proceeded to evaluate their catalytic performance; the HER performance of FeSe_2_-BiSe_2_-CoSe_2_, BiSe_2_, CoSe_2_, FeSe_2_, and Pt/C was characterized in 0.5 M H_2_SO_4_. All electrochemical testing steps were conducted at room temperature. The experimental setup is shown in Figure S4; white wire connects to the reference electrode (Ag/AgCl), red wire connects to the counter electrode (carbon rod), and green wire connects to the working electrode (FeSe_2_-BiSe_2_-CoSe_2_ loaded onto a 1 × 1 cm^2^ carbon cloth). Figure 4a presents a comparative view of the LSV curves for BiSe_2_, CoSe_2_, FeSe_2_, and FeSe_2_-BiSe_2_-CoSe_2_. The overpotentials for FeSe_2_, BiSe_2_, CoSe_2_, and FeSe_2_-BiSe_2_-CoSe_2_ are 247, 240, 155, and 44 mV, respectively. It can be observed that FeSe_2_-BiSe_2_-CoSe_2_ exhibits favorable HER activity [29], surpassing only Pt/C by 30 mV, but, compared with known catalysts, as shown in Figure S3 and Table S4, FeSe_2_-BiSe_2_-CoSe_2_ also exhibits excellent HER activity in acidic environments [30]. The LSV curve was linearly fitted to obtain the catalyst’s Tafel curve (Figure 4b) for analyzing its acidic HER kinetics. FeSe_2_-BiSe_2_-CoSe_2_ exhibited a Tafel slope of 110.23 mV dec^−1^, outperforming BiSe_2_ (159.67 mV dec^−1^), CoSe_2_ (140.06 mV dec^−1^), and FeSe_2_ (111.44 mV dec^−1^), and being slightly higher than Pt/C at 56.08 mV dec^−1^. The lower Tafel value of FeSe_2_-BiSe_2_-CoSe_2_ indicates a faster chemical reaction rate, further demonstrating its excellent HER reactivity [13]. FeSe_2_-BiSe_2_-CoSe_2_ exhibits a Tafel slope ranging from 40 to 120 mV dec^−1^, which demonstrates that the FeSe_2_-BiSe_2_-CoSe_2_ HER reaction pathway follows the Volmer–Heyrovsky mechanism. In 0.5 M H_2_SO_4_, the external circuit transfers electrons to the active sites on the FeSe_2_-BiSe_2_-CoSe_2_ surface, where they combine with H_3_O^+^ in the electrolyte to undergo the Volmer reaction, yielding H_2_O and H* (‘*’ is the active site). Under low H* coverage conditions, H* undergoes the Heyrovsky reaction with the H_3_O^+^ ions and electrons in the solution, yielding H_2_O and H_2_.

The R_ct_ values of BiSe_2_, CoSe_2_, FeSe_2_, and FeSe_2_-BiSe_2_-CoSe_2_ are 8.16, 3.40, 2.95, and 0.63 Ω, respectively (Figure 4c). Among these, FeSe_2_-BiSe_2_-CoSe_2_ exhibits the smallest R_ct_ value compared to BiSe_2_, CoSe_2_, and FeSe_2_, indicating its faster charge transfer rate. This further demonstrates that alloying accelerates the reaction kinetics of the HER [31]. Figure S4 shows the CV curves. In Figure 4d, the measured C_dl_ for FeSe_2_-BiSe_2_-CoSe_2_ was 17.68 mF cm^−2^, substantially exceeding the values recorded for BiSe_2_ (8.08 mF cm^−2^), CoSe_2_ (15.63 mF cm^−2^), and FeSe_2_ (7.74 mF cm^−2^). FeSe_2_-BiSe_2_-CoSe_2_ exhibits an ECSA of 442.0 cm^2^, while BiSe_2_, CoSe_2_, and FeSe_2_ have ECSA values of 202.0 cm^2^, 390.75 cm^2^, and 193.50 cm^2^ (Table S5). These results indicate that FeSe_2_-BiSe_2_-CoSe_2_ possesses a greater ECSA, providing more catalytic active sites for the HER and enhancing the chemical reaction rate. When calculating the TOF value, we consider all atoms in the catalyst to be active sites [32]. Table S6 shows that the TOF value for FeSe_2_-BiSe_2_-CoSe_2_ (2.2 × 10^−2^ s^−1^) is the highest, surpassing that of the CoSe_2_ (2.4 × 10^−3^ s^−1^), FeSe_2_ (1.9 × 10^−2^ s^−1^), and BiSe_2_ (1.8 × 10^−2^ s^−1^). Figure 4e shows a performance comparison of the HER in acidic electrolytes for BiSe_2_, CoSe_2_, FeSe_2_, and FeSe_2_-BiSe_2_-CoSe_2_; FeSe_2_-BiSe_2_-CoSe_2_ exhibits outstanding performance across four HER metrics: overpotential (44 mV), Tafel slope (110.23 mV dec^−1^), C_dl_ value (17.68 mF cm^−2^), and R_ct_ (0.63 Ω). This demonstrates that the FeSe_2_-BiSe_2_-CoSe_2_ ternary heterojunction exhibits significantly superior HER performance in acidic environments compared to BiSe_2_, CoSe_2_, and FeSe_2_ [33]. As shown in Figure 4f, during the 35 h stability test, the potential change in FeSe_2_-BiSe_2_-CoSe_2_ exhibited a trend of initially slow decay followed by stabilization. The LSV curve of FeSe_2_-BiSe_2_-CoSe_2_ revealed negligible overpotential variation, fully confirming the excellent stability of FeSe_2_-BiSe_2_-CoSe_2_, even under strong acidic conditions [34,35].

### 3.3. HER Performance in 1 M KOH

**Figure 5:**
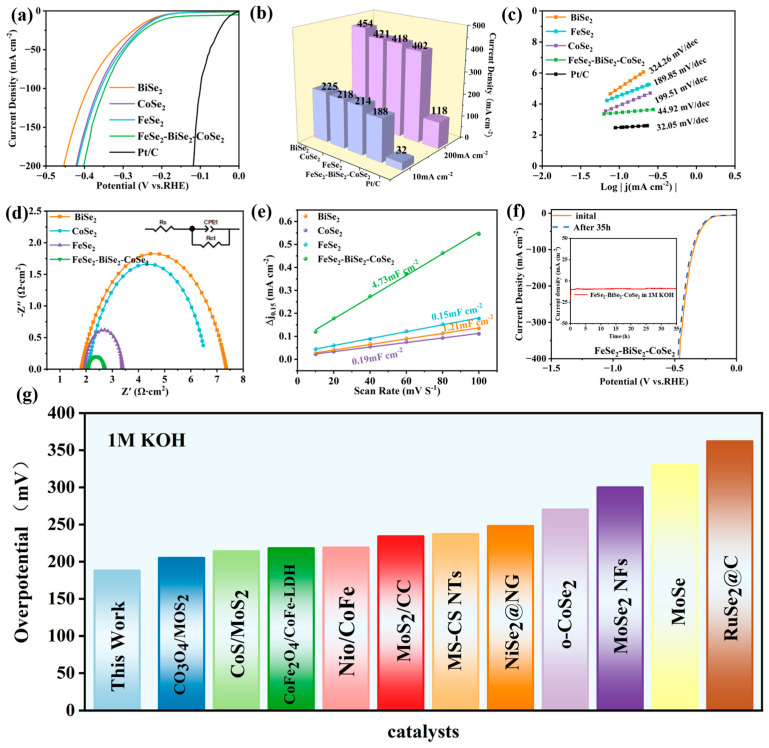
HER performance of BiSe_2_, CoSe_2_, FeSe_2_, and FeSe_2_-BiSe_2_-CoSe_2_ in 1 M KOH. (**a**) LSV curve, (**b**) overpotentials at 10 mA cm^−2^ and 200 mA cm^−2^, (**c**) Tafel slope, (**d**) Nyquist plot, (**e**) C_dl_ curve, (**f**) LSV curve of FeSe_2_-BiSe_2_-CoSe_2_ after 35 h operation in 0.5 M H_2_SO_4_ and i-t curve during continuous 35 h electrolysis (inset), and (**g**) catalyst comparison bar chart.

Using the same testing methodology, the HER performance of BiSe_2_, CoSe_2_, FeSe_2_, Pt/C, and FeSe_2_-BiSe_2_-CoSe_2_ was evaluated in an alkaline environment (1 M KOH). The LSV curves for Pt/C, BiSe_2_, CoSe_2_, FeSe_2_, and FeSe_2_-BiSe_2_-CoSe_2_ are shown in Figure 5a. At a current density of 10 and 200 mA cm^−2^, FeSe_2_-BiSe_2_-CoSe_2_ exhibits an overpotential of 188 and 402 mV, demonstrating superior HER performance compared to BiSe_2_ (225 and 454 mV), CoSe_2_ (218 and 421 mV), and FeSe_2_ (214 and 418 mV), only higher than that of Pt/C at the same current density [36]. Figure 5b shows the overpotentials of Pt/C, BiSe_2_, CoSe_2_, FeSe_2_, and FeSe_2_-BiSe_2_-CoSe_2_ at 10 and 200 mA cm^−2^ under alkaline conditions, indicating that FeSe_2_-BiSe_2_-CoSe_2_ exhibits excellent HER performance in alkaline environments. The Tafel plots derived from the LSV data are depicted in Figure 5c. The Tafel slope of FeSe_2_-BiSe_2_-CoSe_2_ (44.92 mV dec^−1^) is higher than Pt/C at 32.05 mV dec^−1^ but markedly lower than those of BiSe_2_ (324.26 mV dec^−1^), CoSe_2_ (199.51 mV dec^−1^), and FeSe_2_ (189.85 mV dec^−1^). The Tafel slope values of FeSe_2_-BiSe_2_-CoSe_2_ ranging between 40 and 120 mV dec^−1^ indicate adherence to the Volmer–Heyrovsky mechanism [37]. In alkaline media, H_2_O first adsorbs onto active sites on the FeSe_2_-BiSe_2_-CoSe_2_ surface, undergoing the Volmer reaction with electrons to be reduced into H* and OH^−^. Under conditions of low H* coverage, H* undergoes the Heyrovsky reaction with the H_2_O and electrons in the solution to form OH^−^ and H_2_ [38]. The Heyrovsky step plays a dominant role. Furthermore, the R_ct_ values for BiSe_2_, CoSe_2_, FeSe_2_, and FeSe_2_-BiSe_2_-CoSe_2_ were 5.54, 4.38, 1.43, and 0.63 Ω, respectively (Figure 5d). Among these, FeSe_2_-BiSe_2_-CoSe_2_ exhibited the lowest R_ct_ value and minimal charge transfer resistance, indicating that FeSe_2_-BiSe_2_-CoSe_2_ possesses a relatively rapid electron transfer rate in alkaline environments [39].

The CV curves for BiSe_2_, CoSe_2_, FeSe_2_, and FeSe_2_-BiSe_2_-CoSe_2_ were measured within the potential range of 0.3–0.9 V and at scan rates of 10–100 mV s^−1^ (Figure S6). The C_dl_ values for the four materials were obtained (Figure 5e), with FeSe_2_-BiSe_2_-CoSe_2_ exhibiting the highest C_dl_ value of 4.73 mF cm^−2^, significantly exceeding those of BiSe_2_ (1.21 mF cm^−2^), CoSe_2_ (0.19 mF cm^−2^), and FeSe_2_ (0.15 mF cm^−2^) [40]. Calculations of the ECSA based on C_dl_ values (Table S7) reveal that the electrochemical active areas for BiSe_2_, CoSe_2_, FeSe_2_, and FeSe_2_-BiSe_2_-CoSe_2_ are 30.25 cm^2^, 4.75 cm^2^, 3.75 cm^2^, and 118.25 cm^2^, respectively. This demonstrates that FeSe_2_-BiSe_2_-CoSe_2_ possesses the largest electrochemical active area. This indicates that the interface contact area between FeSe_2_-BiSe_2_-CoSe_2_ and the electrolyte is substantial, thereby providing more active sites for interaction with H_2_O. This further accelerates the HER reaction rate under alkaline conditions [33].

Calculations of the TOF for BiSe_2_, CoSe_2_, FeSe_2_, and FeSe_2_-BiSe_2_-CoSe_2_ yielded a TOF of 2.1 × 10^−2^ s^−1^ for FeSe_2_-BiSe_2_-CoSe_2_ (Table S8), significantly higher than that of BiSe_2_, CoSe_2_, and FeSe_2_. This indicates that FeSe_2_-BiSe_2_-CoSe_2_ exhibits superior intrinsic activity [41]. Furthermore, the i-t curve of FeSe_2_-BiSe_2_-CoSe_2_ exhibited negligible current decay after 35 h of continuous testing at a constant potential of 10 mA cm^−2^ (Figure 5f) [42]. The LSV curves before and after the 35 h stability test exhibit near-perfect overlap, confirming FeSe_2_-BiSe_2_-CoSe_2_’s outstanding structural stability and catalytic durability [43,44]. Compared with the previously reported literature, FeSe_2_-BiSe_2_-CoSe_2_ also exhibits relatively superior HER performance in alkaline environments (Figure 5g and Table S4). In summary, FeSe_2_-BiSe_2_-CoSe_2_ demonstrates excellent electrochemical activity and stability under both acidic and alkaline conditions [45,46].

## 4. Conclusions

This study successfully synthesized the ternary heterojunction FeSe_2_-BiSe_2_-CoSe_2_ via a one-step hydrothermal method. Its unique electronic restructuring mechanism significantly enhances hydrogen evolution performance in both acidic and alkaline media. Owing to the substantial electronegativity difference between Bi and Fe/Co, directional charge transfer occurs within the lattice. This reduces the electron density of Bi and Se, forming electron-deficient matrices, while Fe and Co become electron-rich active centers. Concurrently, efficient hybridization occurs between Fe’s 2p orbitals and Se’s 3d orbitals, while energy-level matching between Co and Fe’s 2p orbitals further enhances electron coupling. Collectively, this achieves optimized electronic restructuring. The redistributed electrons not only elevate the intrinsic catalytic activity of active sites but also, in conjunction with the material’s abundant pore structure, facilitate electron and reactant transport. Consequently, the compound exhibits outstanding HER performance and stability in both acidic and alkaline environments.

## Figures and Tables

**Table 1:** Transition metal-based catalysts comparison.

Catalyst Category	Representative Materials	Principal Advantages	Principal Drawbacks
Two-dimensional transition metal dichalcogenide	MoS_2_	High intrinsic activity, high stability	Poor electrical conductivity, limited active sites
Two-dimensional transition metal MSe_2_	MoSe_2_	Highly conductive, intrinsically highly active	Relatively poor stability, difficult to prepare
Transition metal oxides	MnO_2_	Non-precious metals are low in cost and diverse in variety.	Low specific surface area, poor electrical conductivity
Transition metal nitrides	MoN	Excellent electrical conductivity, high stability	The surface is prone to oxidation and the high-temperature synthesis conditions are demanding.
Transition metal phosphides	Ni_2_P	Excellent electrical conductivity, high HER activity	Phosphorus readily leaches or oxidizes during reactions

## Data Availability

The original contributions presented in this study are included in the article/Supplementary Material. Further inquiries can be directed to the corresponding authors.

## Supplementary Materials

The following supporting information can be downloaded at: https://www.mdpi.com/article/10.3390/ma19020430/s1, Figure S1: Full XPS spectra of FeSe_2_-BiSe_2_-CoSe_2_, CoSe_2_, FeSe_2_, and BiSe_2_; Figure S2: Full XPS spectra of (a) FeSe_2_-BiSe_2_-CoSe_2_, (b) FeSe_2_, (c) BiSe_2_, and (d) CoSe_2_; Figure S3: SEM images of (a,b) BiSe_2_, (c,d) CoSe_2_, (e,f) FeSe_2_, and (g,h) FeSe_2_-BiSe_2_-CoSe_2_; Figure S4: Schematic diagram of a three-electrode HER system; Figure S5: Comparison of the HER overpotentials at 10 mA cm^−2^ in 0.5 M H_2_SO_4_; Figure S6: HER cyclic voltammetry of (a) BiSe_2_, (b) CoSe_2_, (c) FeSe_2_, and (d) FeSe_2_-BiSe_2_-CoSe_2_ in 0.5M H_2_SO_4_ at scan rates of 10, 20, 40, 60, 80, and 100 mV s^−1^, respectively; Figure S7: HER cyclic voltammetry of (a) BiSe_2_, (b) CoSe_2_, (c) FeSe_2_, and (d) FeSe_2_-BiSe_2_-CoSe_2_ in 1M KOH at scan rates of 10, 20, 40, 60, 80, and 100 mV s^−1^, respectively; Table S1: The lattice constants were calculated by FeSe_2_ (JCPDS#12-0291, a = 4.815 Å, b = 5.808 Å, and c = 3.599 Å); Table S2: The lattice constants were calculated by BiSe_2_ (JCPDS #12-0732; a = b = 4.133 Å and c = 28.620 Å); Table S3: The lattice constants were calculated by CoSe_2_ (JCPDS #09-0234; a = b = c = 5.858 Å); Table S4: Electrochemical HER performance of the present work compared with previous reports; Table S5: ECSA value of HER in 0.5 M H_2_SO_4_; Table S6: TOF value from HER at the overpotential of −0.35V vs. RHE for catalytic materials_._ (0.5M H_2_SO_4_); Table S7: ECSA value of HER in 1 M KOH; Table S8: TOF value from HER at the overpotential of −0.35V vs. RHE for catalytic materials in 1M KOH). References [47,48,49,50,51,52,53,54,55,56,57,58,59,60,61] are cited in the Supplementary Materials.
