# Supplementary material for: FeSe2-BiSe2-CoSe2 Ternary Heterojunction for Efficient Hydrogen Evolution Reaction Under pH-Universal
Source: Materials (Basel). 2026 Jan 22;19(2):430. doi: 10.3390/ma19020430 (PMC12843173; doi:10.3390/ma19020430)
Supplement: Supplementary file 1 [file materials-19-00430-s001.zip › materials-4102360-supplementary.pdf]

Supporting Information

Section S1. Chemicals

All reagents and chemicals were of analytical grade purity and required no further purification prior to use. Cobalt (II) nitrate hexahydrate (Co $(NO_3)_2 \cdot 6H_2O$) and cetyl trimethyl ammonium bromide ($C_{19}H_{42}BrN$) were sourced from Saen Chemical Technology (Shanghai) Co., Ltd. (Shanghai, China). Ferric nitrate nonahydrate (Fe $(NO_3)_2 \cdot 9H_2O$), bismuth nitrate pentahydrate (Bi $(NO_3)_2 \cdot 5H_2O$), sodium borohydride ($NaBH_4$), and polyethylene glycol 10000 (HO (CH_2CH_2O) nH) were all obtained from Tianjin Shentai Chemical Reagent Co., Ltd. (Tianjin, China). Anhydrous ethanol was obtained from Tianjin Beichen Fangzheng Reagent Factory (Tianjin, China). Selenium powder (Se) was obtained from Shanghai McLean Biochemical Technology Co., Ltd. (Shanghai, China). All water used in the experiments was deionized water.

Section S2. $FeSe_2$-$BiSe_2$-$CoSe_2$ Preparation

Dissolve 1 mmol $Co(NO_3)_2 \cdot 6H_2O$, 1 mmol Bi $(NO_3)_2 \cdot 5H_2O$, and 1 mmol Fe $(NO_3)_2 \cdot 9H_2O$, along with 10 mg polyethylene glycol 10000 and 10 mg hexadecyltrimethylammonium bromide, in 15 mL purified water and 15 mL anhydrous ethanol to obtain Solution A. Dissolve 4 mmol Se and 12 mmol $NaBH_4$ in 15 mL purified water and 15 mL anhydrous ethanol to obtain Solution B. Homogeneously mix Solution A with Solution B, transfer to a 100 mL autoclave lined with PTFE, and react at 200 °C for 16 h. After natural cooling to room temperature, the product was collected by centrifugation, purified several times with water and ethanol, and dried overnight at 60 °C in an oven. The resulting black powder was

identified as $FeSe_2$-$BiSe_2$-$CoSe_2$. As a comparative experiment, $BiSe_2$, $CoSe_2$, and $FeSe_2$ were synthesized under similar experimental conditions. Adding only Bi $(NO_3)_2·5H_2O$ yielded $BiSe_2$, adding only Co $(NO_3)_2·6H_2O$ yielded $CoSe_2$, and adding only Fe $(NO_3)_2·9H_2O$ yielded $FeSe_2$.

Section S3. Characterization of materials

The prepared electrocatalyst was characterised using a Rigaku Miniflex 600 X-ray diffractometer. Testing employed a Cu Kα target as the radiation source (λ = 0.15418 nm), with diffraction scans conducted over 10°-90° at a scan rate of 5° min^{-1}. Samples were tested using an ESCALAB 250Xi XPS system (Thermo Scientific, USA), employing Kα radiation as the excitation source. All data were calibrated against the C1s standard peak (284.8 eV). Microstructural analysis was performed using a Czech TESCAN MIRA LMS SEM under conditions of 10.00 kV acceleration voltage and 9.6 mm working distance. Transmission electron microscopy (TEM, JEM2100PLUS) and high-resolution transmission electron microscopy (HRTEM, JEM2100PLUS) were employed to analyze the microstructure and structural information of the materials. Electron micrographs were utilized to examine lattice fringes within the HRTEM images. Elemental distribution was recorded via high-angle annular dark-field scanning transmission electron microscopy (HAADF-STEM, JEM-F200(HRP)) and X-ray energy dispersive spectroscopy (EDX, JEM-F200(HRP)) elemental mapping.

Section S4. Calculation of Lattice Constants by XRD Patterns

By measuring the diffraction angle (theta) and the X-ray with known

wavelength(lambda), the Bragg 's law is used to calculate the lattice plane spacing (d) according to the Bragg equation: $d = n\times\lambda/2\times\sin\theta$, where θ is the incident line, the angle between the reflection line and the reflection crystal plane (measured), the wavelength is 1.5406 Å, and n is the reflection series 2. Then, according to the measurement of different lattice plane spacing, and the calculation formula $d=a/\ (4/3)\times(h^2+k^2+hk)+l^2\times(a^2/c^2))^{1/2}$ ($FeSe_2$) and $1/d^2=(h^2+k^2)/a^2+l^2/c^2$($BiSe_2$) to determine the lattice constant.

Section S5. Electrochemical Measurements

Working electrode preparation: The synthesized catalyst was loaded onto a 1×1 cm^2 foam nickel electrode to serve as the working electrode. To prepare the test sample, 10 mg of catalyst was dispersed in 450 μL of anhydrous ethanol and 50 μL of Nafion solution (5 wt%), followed by ultrasonication for 2 hours. A 100 μL aliquot of the homogenized catalyst solution was uniformly coated onto both sides of the foam nickel (1.0 × 1.0 cm^2). The electrode sheets were then air-dried at room temperature.

Acidic HER Performance Testing: This work employed a standard three-electrode system within an electrochemical workstation (CHI660E), utilizing 0.5 M H_2SO_4 as the electrolyte, with a graphite rod and Ag/AgCl electrode serving as the counter and reference electrodes respectively. Prior to measurement, cyclic voltammetry (CV) activation testing was performed at a scan rate of 100 mV s^{-1} between -0.25 V and -0.45 V. Electrochemical impedance spectroscopy (EIS) was performed over a frequency range of 10^5 to 10^{-2} Hz. Linear sweep voltammetry (LSV) was recorded at a scan rate of 5 mV s^{-1} across the potential range of 0 V to -2 V.

Overpotential versus logarithm of current density (η versus log j) plots were generated from the LSV curves to derive Tafel plots for evaluating HER kinetics in the alkaline electrolyte. Cyclic voltammetry (CV) was recorded at scan rates of 10, 20, 40, 60, 80, and 100 mV s−1 to evaluate double-layer capacitance (C_{dl}). For stability testing of HER performance, continuous electrolysis was conducted for 35 hours using the time-current method (it).

Alkaline HER performance testing: This work employed a standard three-electrode system within an electrochemical workstation (CHI660E). The electrolyte comprised 1 M KOH solution, with a graphite rod serving as the working electrode and a Hg/HgO electrode (in 1 M KOH electrolyte) as the reference electrode. All other testing parameters remained consistent with the HER testing under acidic conditions. All measurements were corrected according to the reversible hydrogen electrode (RHE) according to the formula S1 and S2.

$$E(\text{vs. RHE}) = E(\text{vs. Hg/HgO}) + 0.098 + 0.0592 \times \text{pH} \qquad \text{(S1)}$$

$$E(\text{vs. RHE}) = E(\text{vs. Ag/AgCl}) + 0.197 + 0.0592 \times \text{pH} \qquad \text{(S2)}$$

Section S6. ECSA calculation

Electrochemical capacitance measurements were used to determine the active surface area of each catalyst. To measure the electrochemical capacitance, the potential was swept between -0.10 to -0.30 V vs Ag/AgCl 8 times at each of eight different scan rates (10, 20, 40, 60, 80, 100 mV/s). Specific capacitance values for 1 cm^{-2} flat standards were used and the specific capacitance was converted to electrochemically active surface area (ECSA) using Equation S3.

$$ECSA = C_{dl}/C_s \qquad (S3)$$

The specific capacitance for a fat surface was generally found to be in the range of 20-60 μF cm^{-2}. In the following calculations, we assumed 40 μF cm^{-2}.

The electrolyte is 0.5M H_2SO_4.

$FeSe_2$-$BiSe_2$-$CoSe_2$:

$$A_{ECSA} = \frac{17.68\ F.cm^{-2}}{40\mu F.cm^{-2} per\ cm^2_{ECSA}} = 442.00 cm^2_{ECSA}$$

$BiSe_2$:

$$A_{ECSA} = \frac{8.08\ mF.cm^{-2}}{40\mu F.cm^{-2} per\ cm^2_{ECSA}} = 202.00 cm^2_{ECSA}$$

$CoSe_2$:

$$A_{ECSA} = \frac{15.63\ mF.cm^{-2}}{40\mu F.cm^{-2} per\ cm^2_{ECSA}} = 390.75\ cm^2_{ECSA}$$

$FeSe_2$:

$$A_{ECSA} = \frac{7.74\ mF.cm^{-2}}{40\mu F.cm^{-2} per\ cm^2_{ECSA}} = 193.50\ cm^2_{ECSA}$$

The electrolyte is 1M KOH.

$FeSe_2$-$BiSe_2$-$CoSe_2$:

$$A_{ECSA} = \frac{4.73 F.cm^{-2}}{40\mu F.cm^{-2} per\ cm^2_{ECSA}} = 118.25\ cm^2_{ECSA}$$

$BiSe_2$:

$$A_{ECSA} = \frac{1.21\ mF.cm^{-2}}{40\mu F.cm^{-2} per\ cm^2_{ECSA}} = 30.25\ cm^2_{ECSA}$$

$CoSe_2$:

$$A_{ECSA} = \frac{0.19\ mF.cm^{-2}}{40\mu F.cm^{-2} per\ cm^2_{ECSA}} = 4.75\ cm^2_{ECSA}$$

$FeSe_2$:

$$A_{ECSA} = \frac{0.15\ mF.cm^{-2}}{40\mu F.cm^{-2} per\ cm^2_{ECSA}} = 3.75\ cm^2_{ECSA}$$

Section S7. The Turnover Frequency (TOF) calculation:

The turnover frequency (TOF) was used to estimate the intrinsic activity. The estimated intrinsic activity of TOF can be calculated from the formula.

TOF is calculated as

$$TOF = J \times \frac{S}{(x \times F \times n)}$$

J: current (in A) during the linear sweep measurement.

S: the area of the working electrode.

x: The factor ½ arrives by taking into account that two electrons are required to form one hydrogen.

F: Faraday constant (~ 96485 C mol^{-1}).

n: the number of active sites (mol).

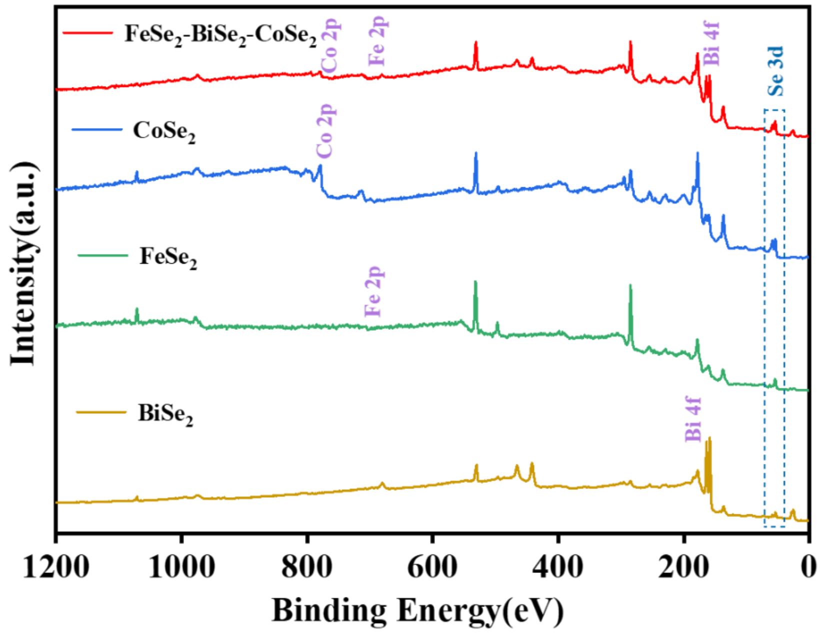

Figure S1 Full XPS spectra of $FeSe_2$-$BiSe_2$-$CoSe_2$, $CoSe_2$, $FeSe_2$, and $BiSe_2$.

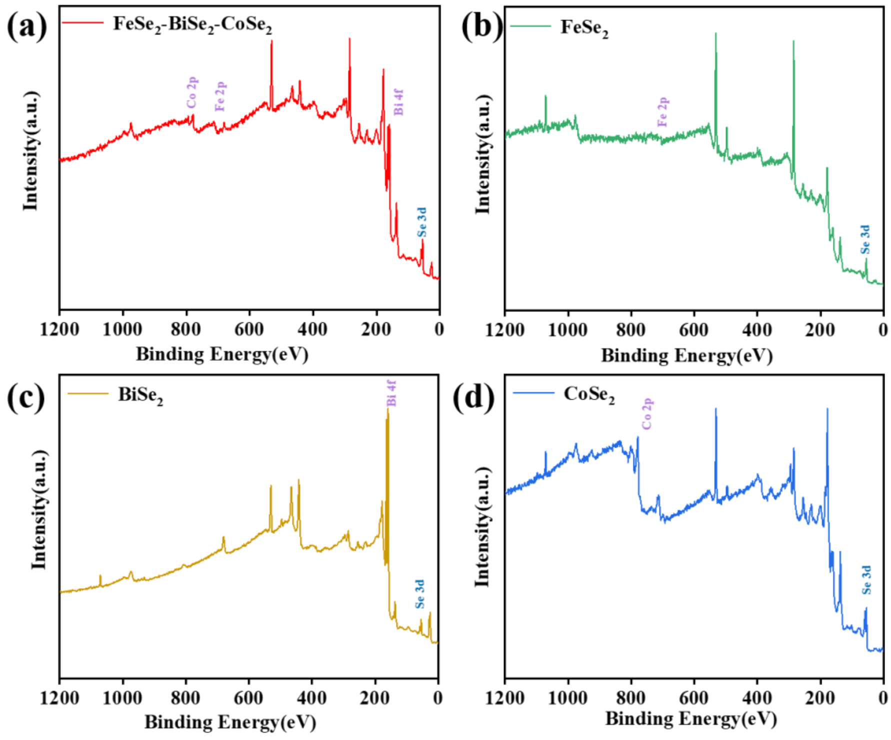

Figure S2 Full XPS spectra of (a) $FeSe_2$-$BiSe_2$-$CoSe_2$, (b) $FeSe_2$, (c) $BiSe_2$, and (d) $CoSe_2$.

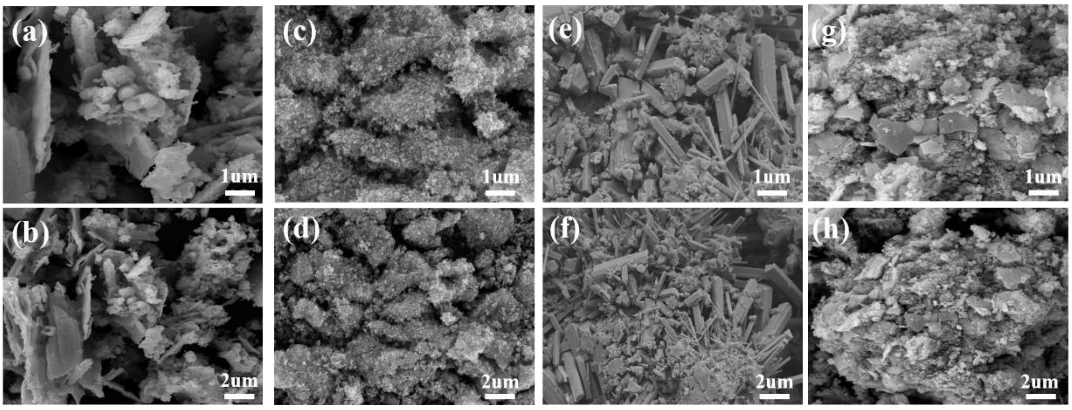

Figure S3. SEM images of (a-b) $BiSe_2$, (c-d) $CoSe_2$, (e-f) $FeSe_2$, and (g-h) $FeSe_2$-$BiSe_2$-$CoSe_2$.

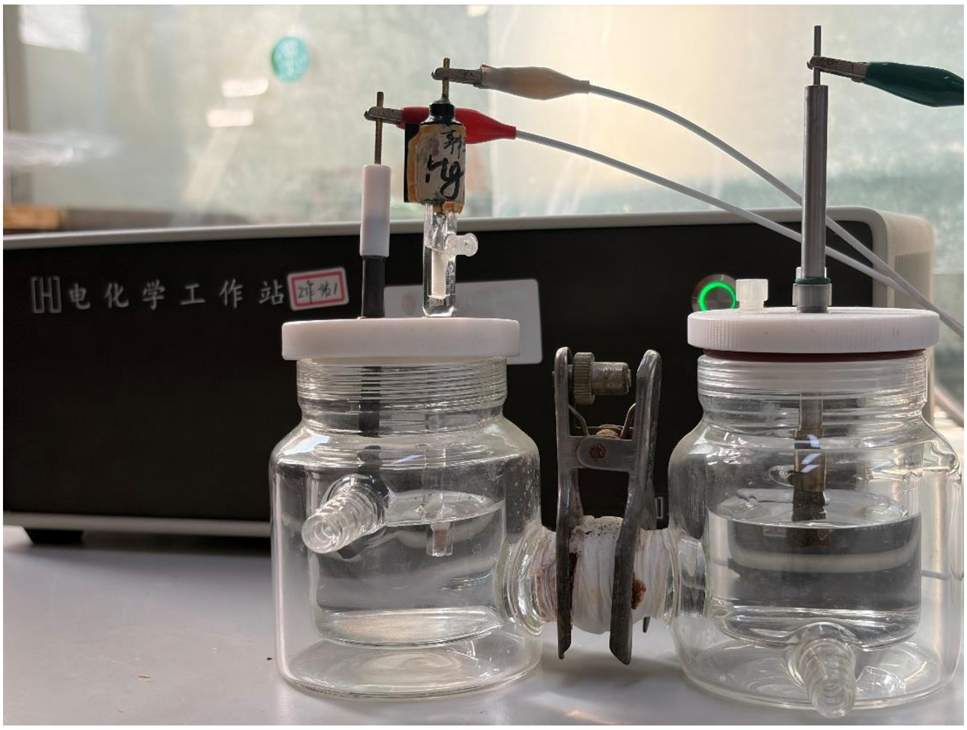

Figure S4. Schematic diagram of a Three-Electrode HER System.

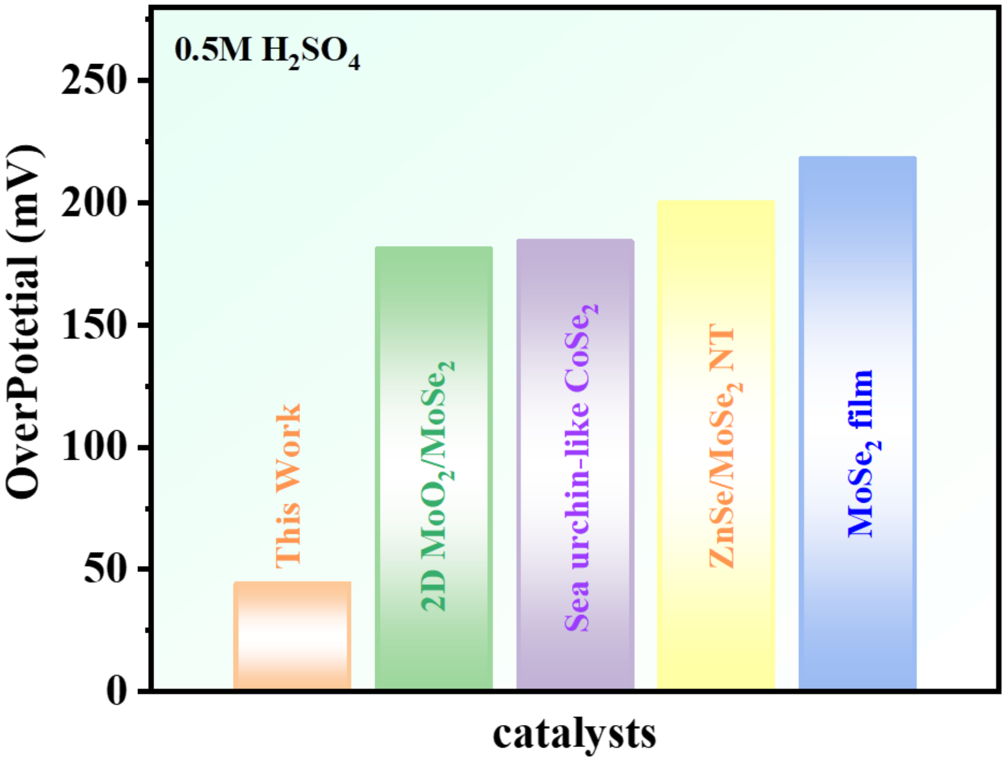

Figure S5 Comparison of the HER overpotentials at 10 mA cm^{-2} in 0.5 M H_2SO_4.

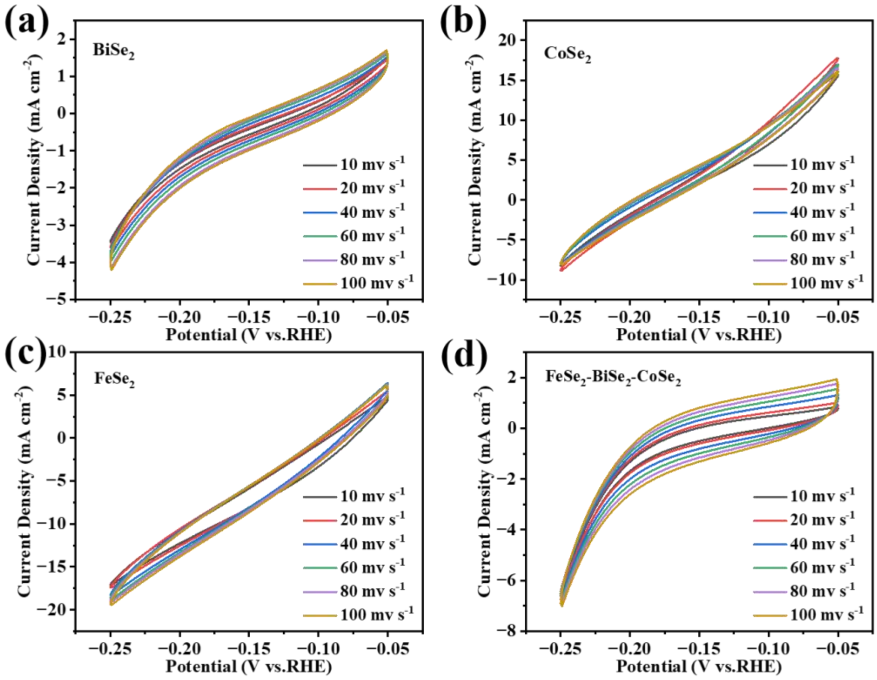

Figure S6 HER cyclic voltammetry of (a) $BiSe_2$, (b) $CoSe_2$, (c) $FeSe_2$ and (d) $FeSe_2$-$BiSe_2$-$CoSe_2$ in 0.5M H_2SO_4 at scan rates of 10, 20, 40, 60, 80 and 100 mV s^{-1}, respectively.

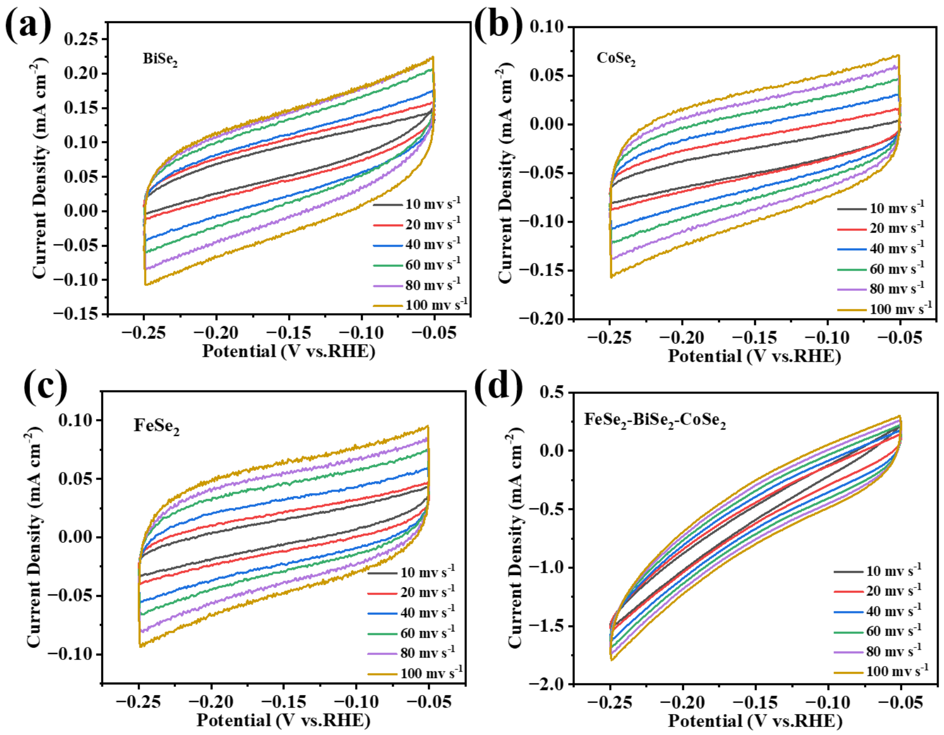

Figure S7 HER cyclic voltammetry of (a) $BiSe_2$, (b) $CoSe_2$, (c) $FeSe_2$ and (d) $FeSe_2$-$BiSe_2$-$CoSe_2$. in 1M KOH at scan rates of 10, 20, 40, 60, 80 and 100 mV s^{-1}, respectively.

Table S1 The lattice constants were calculated by $FeSe_2$ (JCPDS#12-0291, a=4.815 Å, b=5.808 Å, c=3.599 Å).

	NO.	Peak Position 2 Theta (°)	Interplanar spacing d (Å)	Miller indices (h k l)	Lattice constant
	1	30.21	2.94	(0 2 0)	a =4.857Å
$FeSe_2$	2	35.01	2.58	(1 1 1)	b=5.876 Å
	3	37.03	2.41	(2 0 0)	c=3.587 Å

	4	50.83	1.79	（0 0 2）
	5	55.69	1.64	（2 2 1）
$FeSe_2$-$BiSe_2$-$CoSe_2$	1	34.89	2.58	（1 1 1）
	2	36.22	2.49	（1 2 0）
	3	48.12	1.89	（2 1 1）

Table S2 The lattice constants were calculated by $BiSe_2$ (JCPDS #12-0732; a=b=4.133 Å, c=28.620 Å).

	NO.	Peak Position 2 Theta (°)	Interplanar spacing d (Å)	Miller indices (h k l)	Lattice constant
$BiSe_2$	1	29.23	3.03	(0 1 5)	
	2	40.15	2.23	(1 0 1 0)	
	3	43.56	2.07	(1 10)	
	4	53.43	1.71	(2 0 5)	a=b=4.151 Å c=28.666 Å
	5	66.54	1.40	(1 1 1 5)	
$FeSe_2$-$BiSe_2$-$CoSe_2$	1	29.35	3.03	(0 1 5)	
	2	40.44	2.23	(1 0 1 0)	
	3	43.73	2.07	(1 1 0)	

Table S3 The lattice constants were calculated by $CoSe_2$ (JCPDS #09-0234; a=b=c=5.858 Å).

	NO.	Peak Position 2 Theta (°)	Interplanar spacing d (Å)	Miller indices (h k l)	Lattice constant
$CoSe_2$	1	33.74	2.62	(2 1 0)	
	2	45.87	1.95	(2 2 1)	
	3	51.35	1.76	(3 1 1)	
	4	62.91	1.46	(4 0 0)	a=b=c=5.874 Å
$FeSe_2$-$BiSe_2$-$CoSe_2$	1	51.01	1.76	（3 1 1）	
	2	53.78	1.69	（2 2 2）	
	3	71.51	1.31	（4 2 0）	

Table S4 Electrochemical HER performance of the present work compared with previous reports.

Catalyst	Electrolyte	Over Potential(mV), η at 10 mA cm^{-2}	Ref.
$FeSe_2$-$BiSe_2$-$CoSe_2$	1M KOH	188	This work
MoS_2/CC	1M KOH	234	[1]
$RuSe_2$@C	1M KOH	362	[2]
$CoFe_2O_4$/CoFe-LDH	1M KOH	218	[3]
NiO/CoFe	1M KOH	219	[4]
MS-CS NTs	1M KOH	237	[5]
CoS/MoS_2	1M KOH	214	[6]
CO_3O_4/MOS_2	1M KOH	205	[7]
$MoSe_2$ NFs	1M KOH	300	[8]
$NiSe_2$@NG	1M KOH	248	[9]
MoSe	1M KOH	331	[10]
o-$CoSe_2$	1M KOH	270	[11]
$FeSe_2$-$BiSe_2$-$CoSe_2$	0.5M H_2SO_4	44	This work
Sea urchin-like $CoSe_2$	0.5 M H_2SO_4	184	[12]
ZnSe/$MoSe_2$ NT	0.5 M H_2SO_4	200	[13]
$MoSe_2$ film	0.5 M H_2SO_4	218	[14]
2D MoO_2/$MoSe_2$	1.0 M H_2SO_4	181	[15]

Table S5 ECSA value of HER in 0.5 M H_2SO_4

Sample	ECSA (cm_{ECSA}^2)
$CoSe_2$	390.75
$BiSe_2$	202.00
$FeSe_2$	193.50
$FeSe_2$-$BiSe_2$-$CoSe_2$	442.00

Table S6 TOF value from HER at the overpotential of -0.35V vs RHE for catalytic materials. (0.5M H_2SO_4)

Sample	Current density at -0.35V vs RHE (mV)	n (mol)	TOF (s^{-1})
$FeSe_2$-$BiSe_2$-$CoSe_2$	110.58	0.025592952	2.20×10^{-2}
$CoSe_2$	79.02	0.172637873	2.40×10^{-3}
$BiSe_2$	61.80	0.018695109	1.70×10^{-2}
$FeSe_2$	99.10	0.027270402	1.90×10^{-2}

Table S7 ECSA value of HER in 1 M KOH

Sample	ECSA (cm_{ECSA}^2)
$CoSe_2$	4.75
$BiSe_2$	30.25
$FeSe_2$	3.75
$FeSe_2$-$BiSe_2$-$CoSe_2$	118.25

Table S8 TOF value from HER at the overpotential of -0.35V vs RHE for catalytic materials in 1M KOH)

Sample	Current density at 0.35V vs RHE (mV)	n (mol)	TOF (s^{-1})
$FeSe_2$-$BiSe_2$-$CoSe_2$	103.00	0.025592952	2.10×10^{-2}
$CoSe_2$	83.50	0.172637873	2.50×10^{-3}
$BiSe_2$	64.24	0.018695109	1.80×10^{-2}
$FeSe_2$	90.50	0.027270402	1.70×10^{-2}

References

1. Song, M.; Zhao, Y.; Wu, Z.X.; Liu, X.E. MoS_2/CoB with Se doping on carbon cloth to drive overall water-splitting in an alkaline electrolyte. Sustain. Energ. Fuels 2020, 4, 5036-41.

2. Qin, J.F.; Shen, Q.H.; Du, C.C.; Hong, M.; Yang, Y.X.; Zhang, X.H.; Chen, J.H. Enriched Se vacancies engineering of $RuSe_2$ induced by low-valence Cu doping for promoting hydrogen evolution and coupling power generation. Fuel 2024, 361, 130752.

3. Wang, Y.Q.; Jian, C.Y.; Hong, W.T.; Cai, Q.; Liu, W. Tuning the electron status of urchin-like CoS_2 nanowires by selenium doping toward highly efficient hydrogen evolution reaction. Mater. Lett. 2019, 257, 126673.

4. Lei, Y.T.; Zhang, L.L.; Zhou, D.N.; Xiong, C.L.; Zhao, Y.F.; Chen, W.X.; Xiang, X.; Shang, H.S.; Zhang, B. Construction of interconnected NiO/CoFe alloy nanosheets for overall water splitting. Renew. Energy 2022, 194, 459-68.

5. Su, C.; Xiang, J.Y.; Wen, F.S.; Song, L.Z.; Mu, C.P.; Xu, D.Y.; Hao, C.X.; Liu, Z.Y. Microwave synthesized three-dimensional hierarchical nanostructure CoS_2/MoS_2 growth on carbon fiber cloth: A bifunctional electrode for hydrogen evolution reaction and supercapacitor. Electrochim. Acta 2016, 212, 941-9.

6. Lin, H.F.; Li, H.Y.; Li, Y.Y.; Liu, J.L.; Wang, X.; Wang, L. Hierarchical CoS/MoS2 and $Co_3S_4/MoS_2/Ni_2P$ nanotubes for efficient electrocatalytic hydrogen evolution in alkaline media. J. Mater. Chem. A 2017, 5, 25410-9.

7. Muthurasu, A.; Maruthapandian, V.; Kim, H.Y. Metal-organic framework derived

Co_3O_4/MoS_2 heterostructure for efficient bifunctional electrocatalysts for oxygen evolution reaction and hydrogen evolution reaction. Appl. Catal. B-Environ. Energy 2019, 248, 202-10.

8. Tang, Y.; Yang, C.H.; Sheng, M.H.; Yin, X.T.; Que, W.X. Synergistically coupling phosphorus-doped molybdenum carbide with MXene as a highly efficient and stable electrocatalyst for hydrogen evolution reaction. ACS Sustain. Chem. Eng. 2020, 8, 12990-8.

9. Li, W.X.; Yu, B.; Hu, Y.; Wang, X.Q.; Yang, D.X.; Chen, Y.F. Core-shell structure of $NiSe_2$ nanoparticles@nitrogen-doped graphene for hydrogen evolution reaction in both acidic and alkaline media. ACS Sustain. Chem. Eng. 2019, 7, 20463–73.

10. Zhao, G.Q.; Li, P.; Rui, K.; Chen, Y.P.; Dou, S.X.; Sun, W.P. $CoSe_2/MoSe_2$ heterostructures with enriched water adsorption/dissociation sites towards enhanced alkaline hydrogen evolution reaction. Chem.-Eur. J. 2018, 24, 11158-65.

11. Chen, P.Z.; Xu, K.; Tao, S.; Zhou, T.P.; Tong, Y.; Ding, H.; Zhang, L.D.; Chu, W.S.; Wu, C.Z.; Xie, Y. Phase-transformation engineering in cobalt diselenide realizing enhanced catalytic activity for hydrogen evolution in an alkaline medium. Adv. Mater. 2016, 28, 7527-32.

12. Cheng, L.; Huang, W.J.; Gong, Q.F.; Liu, C.H.; Liu, Z.; Li, Y.G.; Dai, H.J. Ultrathin WS_2 nanoflakes as a high-performance electrocatalyst for the hydrogen evolution reaction. Angew. Chem.-Int. Edit. 2014, 53, 7860-3.

13. Liang, H.W.; Brüller, S.; Dong, R.H.; Zhang, J.; Feng, X.L.; Müllen, K. Molecular metal-N_x centres in porous carbon for electrocatalytic hydrogen evolution.

Nat. Commun. 2015, 6, 8992.

14. Kang, W.J.; Feng, Y.; Li, Z.; Yang, W.Q.; Cheng, C.Q.; Shi, Z.Z.; Yin, P.F.; Shen, G.R.; Yang, J.; Dong, C.K.; et al. Strain-activated copper catalyst for pH-Universal hydrogen evolution reaction. Adv. Funct. Mater. 2022, 2112367.

15. Jiang, L.L.; Ji, S.J.; Xue, H.G.; Suen, N.T. HER activity of M_xNi_{1-x} (M = Cr, Mo and W; x ≈ 0.2) alloy in acid and alkaline media. Int. J. Hydrog. Energy 2020, 45, 17533-9.
